# Antioxidant Activity of the Extracts of Some Cowpea (*Vigna unguiculata* (L) Walp.) Cultivars Commonly Consumed in Pakistan

**DOI:** 10.3390/molecules18022005

**Published:** 2013-02-05

**Authors:** Muhammad Zia-Ul-Haq, Shakeel Ahmad, Ryszard Amarowicz, Vincenzo De Feo

**Affiliations:** 1 Department of Pharmacognosy, University of Karachi, Karachi-75270, Pakistan; E-Mail: ahirzia@gmail.com; 2 Department of Agronomy, Bahauddin Zakariya University, Multan-60800, Pakistan; E-Mail: shakeel.agronomy@gmail.com; 3 Institute of Animal Reproduction and Food Research of the Polish Academy of Sciences, Tuwima Str. 10, 10-748 Olsztyn, Poland; 4 Department of Pharmaceutical and Biomedical Sciences, Salerno University, Fisciano, 84084 Salerno, Italy; E-Mail: defeo@unisa.it

**Keywords:** cowpea, antioxidant potential, phenolic compounds, Pakistan

## Abstract

The present investigation has been carried out to determine the antioxidant activity of the methanolic extracts obtained from four cultivars of cowpea (*Vigna unguiculata* (L) Walp.) seeds. Phenolic compounds present in the extracts showed the antioxidant and antiradical properties when investigated using a linoleic acid peroxidation model, FRAP, ORAC and TRAP assays, as well as DPPH, hydroxyl, nitric oxide and superoxide radical scavenging activity. The HPLC analysis of the cowpea extracts showed the presence of neochlorogenic acid, chlorogenic acid and caffeic acids. The results indicated that methanolic extract of the cowpea resembled in the aforementioned activities those from other leguminous seeds and pulses. Phenolic constituents contained in cowpea may have a future role as ingredients in the development of functional foods.

## 1. Introduction

Cowpea (*Vigna unguiculata* (L) Walp.) is one of the most ancient food sources and has probably been used as a crop plant since Neolithic times [1]. Like for many other legumes, its seeds are the most economically valuable plant part of cowpea and are well-known due to their ascribed nutritional and medicinal properties. Known to be an excellent source of protein, cowpea is also rich in important vitamins, minerals, and soluble and insoluble dietary fiber. All parts of cowpea plants are used for food or fodder. The tender shoot tips and leaves are consumed when they reach the seeding stage while immature pods and seeds are consumed during the fruiting stage. Harvested dry seeds can be ground into a slurry to make cowpea cake, or deep fried into bean balls, or the seeds could be boiled, mixed with sauce or stew and consumed directly. Plant residues are used as fodder for farm animals [2]. Cowpea is used in culinary dishes in the Indo-Pakistan sub-continent. The consumption of cowpea seeds, after processing such as soaking, dry heating, followed by cooking along with cooked rice, is a common cuisine among the rural people in Pakistan. Fresh young leaves, immature pods, and seeds are used as vegetables, while dry grain is used to prepare main meal dishes and snacks [3]. The cooking liquor of the seeds with spices is considered to be a potential remedy for the common cold. Leaves are boiled, drained, sun-dried and then stored for later use. Seed oil exhibit antidiabetic properties [4]. Seeds possess nematicidal and antifungal properties [5]. Green cowpea seeds are sometimes roasted like peanuts. The roots are eaten in Sudan and Ethiopia. Scorched seeds are occasionally used as a coffee substitute. The seed is diuretic and after eating after boiling is considered to destroy worms in the stomach [6].

In Pakistan, various varieties of cowpeas are popularly consumed as a source of dietary proteins, while the cultivation and consumption of cowpea is increasing due to recent reports on the their high nutritional qualities. Although some preliminary information has been presented in an earlier report [7], detailed investigations are still required as no report on the antioxidant potential of cowpea indigenous to Pakistan has been presented so far. This study has been conducted to determine the antioxidant properties of the extracts of cowpea to explore its beneficial effects as a potential functional food.

## 2. Results and Discussion

Antioxidants are an important part of the defense system of the human body and help to cope with oxidative stress caused by reactive oxygen species [8]. There is a growing interest in the antioxidant activity of phenolics and condensed tannin contents of plant extracts due to their potential role in disease prevention and health promotion. Estimation of total phenolic contents (TPC) and condensed tannin contents is a common-bench assay and first step used during evaluation of antioxidant activity of plant extracts and natural products isolated therefrom. TPC values (expressed in mg GAE/g) of cowpea seed extracts are presented in Table 1. A wide variation was observed for phenolic contents and the cultivars differed significantly with respect to this parameter. The highest TPC was obtained in the case of White star (19.3 mg GAE/g) whereas the lowest TPC was obtained in the case of CP1 (11.9 mg GAE/g). Our values for total phenolic contents are in partial agreement with those reported by other authors [9] for cowpea. Phenolic contents are comparatively greater than those observed for seed extracts of chickpea and lentil cultivars from Pakistan [10,11]. According to literature data, the total phenolic content is directly associated with antioxidant activity [12,13]. Highest condensed tannin contents were observed for White star (25.4 mg CE/g) while lowest was observed for CP1 (14.9 mg CE/g). It is evident that condensed tannin contents are also greater than those of seed extracts of chickpea and lentil from Pakistan. Condensed tannins are located mainly in the testa and play an important role in the defense system of seeds that are exposed to oxidative damage by many environmental factors [14]. It is well-known that phenolic content as well as condensed tannin contents vary depending on several factors such as different genotype, growing condition, agronomic practices employed, season, maturity, post-harvest storage and processing conditions and solvent used for extraction. The results indicate that cowpea seeds are rich in antioxidant activity than chickpea and lentil seed as antioxidant activity is dependent to a large extent on these constituents. 

**Figure 1:**
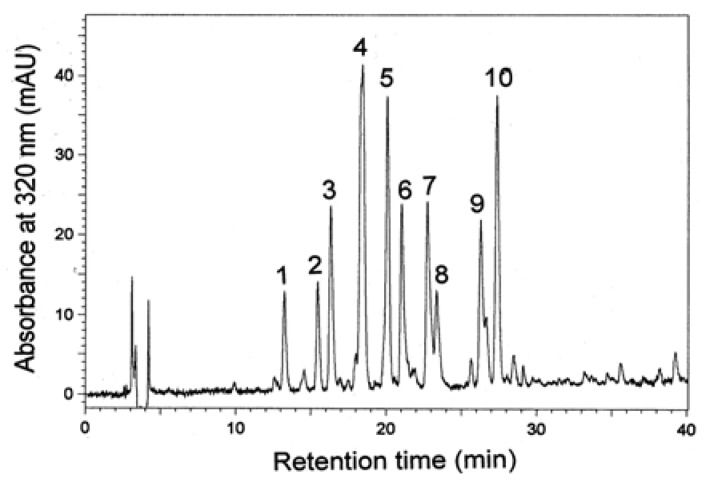
HPLC chromatogram of phenolic compounds of extracts of seeds of White star cowpea cultivar recorded at 320 nm.

**Figure 2:**
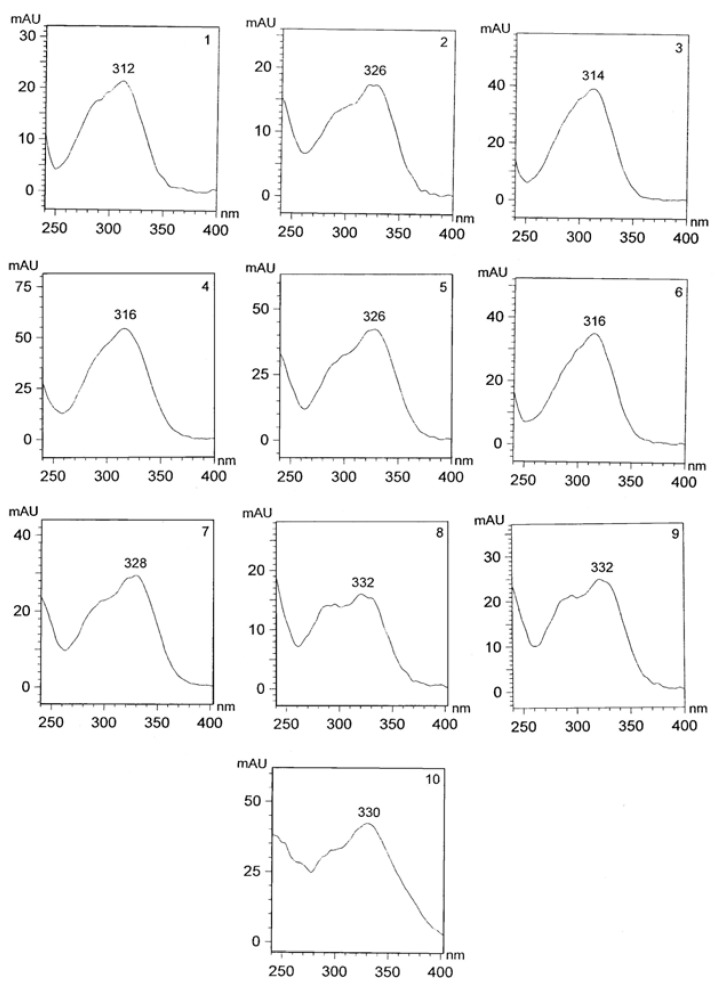
UV-DAD spectra of individual phenolic compounds (numbers of compounds are the same as numbers of peaks mentioned in Figure 1) of extracts of the seeds of White star cowpea cultivar.

Due to the complexity of plant phenolics and different reactivity of phenols toward assay reagents, these results should be reinforced by HPLC analysis, currently the most popular and reliable technique for analysis of phenolic compounds. The RP-HPLC chromatograms of cowpea extracts recorded at 320 nm were characterized by the presence of ten dominant peaks (1–10) with a retention times of 13.1, 15.2, 16.5, 18.6, 20.1, 21.3, 22.9, 23.0, 26.2 and 27.8 min (Figure 1 and Figure 2), respectively. UV spectra of the compounds (peaks 1–10) exhibited maxima at 312, 326, 314, 316, 326, 316, 328, 332, 332 and 330 nm, respectively. Using original standards compounds **2**, **5** and **7** were identified as neochlorogenic, chlorogenic, and caffeic acids. Based on the UV-DAD spectra other compounds were tentatively elucidated as *p*-coumaric and caffeic acid derivatives. The extract of White star was characterized by the highest content of individual phenolic compounds (Table 2). The presence of phenolic acids was reported before for the green and red lentil, chicpea, white bean, adzuki bean, and pea [11,15,16,17,18,19,20]. Phenolic acids noted in cowpea extract are desirable from nutritional point of view because a positive correlation between the consumption of phenolic-rich foods and a decrease of several chronic diseases has been shown to exist from epidemiological studies [21,22].

The DPPH• radial scavenging assay was used to assess the scavenging activity of cowpea seed extracts as shown in Table 3. DPPH• is increasingly used for quickly assessing the ability of antioxidants to transfer the labile H atoms to radicals. The antiradical capacity values of cowpea against DPPH• ranged from 25.1 to 32.5 μmol Trolox/g. Our results are close to those reported earlier for cowpea and other food legumes [9,10,11]. Differences between our results and previous reports may be attributed partly to the differences in the source of materials. The FRAP assay is simple, speedy, inexpensive, and highly reproducible. Antioxidant potential of the cowpea seed extracts was estimated from their ability to reduce TPTZ-Fe^3+^ to TPTZ-Fe^2+^ complex. The FRAP values of the antioxidant extracts from selected cowpea varieties are presented in Table 3. FRAP values of cowpea ranged from 13.2 to 19.4 mmol Fe^2+^/g. Our results are different to those reported earlier [9] perhaps due to different units of expressions. The ORAC method is usually employed to estimate antioxidant activity of foods and to evaluate *in vivo* responses to dietary antioxidant manipulation. The ORAC values ranged from 83.8 to 96.2 µmol Trolox/g and are greater than those observed for desi chickpea and lentil from Pakistan [10,11]. Cowpea extract exhibited good antioxidant activity when assessed by linoleic acid peroxidation system (Table 3) and higher antioxidant activity was observed as compared to chickpea seeds [11]. Antioxidant activity of the extracts of leguminous seeds (pea, bean, lentil, faba bean, broad bean, everlasting bean, and chickpea) in a β-carotene-linoleate model system has been reported in several studies [13,19,23,24,25,26]. Total antioxidant capacity was observed by TRAP assay. The values reported are different from those observed from values reported for seeds of *Capparis deciduas*, *Lepidium sativum* and *Ipomoea hederacea* from Pakistan [8,27]. The high value of TRAP reported by the mentioned authors was probably by the tannins which were dominating phenolic compounds in the investigated seed extracts.

Extracts of seeds were assessed for their potential scavenging antiradical activity against some common radicals like hydroxyl, nitric oxide and superoxide and values expressed as IC_50_ μg/mL. Hydroxyl radical is an extremely reactive free radical formed in biological systems and a highly damaging species in free radical pathology as it may damage almost every molecule found in living cells [28]. This radical may join nucleotides in DNA and cause strand breakage which contributes to carcinogenesis, mutagenesis and cytotoxicity [29]. Hydroxyl radical scavenging capacity of an extract is directly related to its antioxidant activity [30]. The extracts exhibited significant hydroxyl radical scavenging activity (Table 4) with IC_50_ from 80.6 to 92.4 μg/mL for White star and CP1 respectively. Nitric oxide has an unpaired electron, hence is a free radical nitric oxide. Plant extracts and natural products isolated may counteract the ill-effect of nitric oxide *in vivo* [31]. The superoxide radical scavenging assay is said to be more relevant than those methods described above, because it utilizes a biologically relevant radical source. This radical mediates inflammatory tissue injuries in ischemia-reperfusion, arthritis, gout and gastric ulceration. Superoxide radical has a low reactivity and a low capacity to penetrate the lipidic membrane layer, but it can generate hydrogen peroxide and highly reactive hydroxyl radical, via Haber-Weiss reaction [27]. The extracts exhibited significant superoxide radical scavenging activity (Table 4).

According to literature data the antiradical activities of leguminous extracts were investigated using an EPR spin trapping method [32] enhanced chemiluminescence and photoluminescence [33,34], scavenging of AMVN radical [35], ABAP radical [36], DPPH radical [15,16,17,37], ABTS cation radical [15,16,17,37], superoxide anion radical [38] peroxyl radical [36,37].

## 3. Experimental

### 3.1. Plant Material and Chemicals

Seeds of four cowpea cultivars CP1, CP2, White star and AS dandy (1 kg for each cultivar) were procured from the Department of Agronomy, Bahauddin Zakariya University, Multan, Pakistan. Seeds were stored in stainless-steel containers at 4 °C prior to analysis. All solvents used were of HPLC or analytical grade unless otherwise specified. Ferric chloride, ferrous chloride, ammonium thiocyanate, deoxyribose, trichloroacetic acid (TCA), (+)-catechin, gallic acid, chlorogenic acid, caffeic acid, *p-*coumaric acid, vanillin, fluorescein, 2,2-azobis(2-amidinopropane) dihydrochloride (ABAP), EDTA, butylated hydroxyanisole (BHA), ascorbic acid, Folin and Ciocalteu’s phenol reagent, 2,2-diphenyl-1-picrylhydrazyl radical (DPPH^•^), 2,2’-azinobis-(3-ethylbenzothiazoline-6-sulfonic acid) (ABTS), 6-hydroxy-2,5,7,8-tetramethyl-chroman-2-carboxylic acid (Trolox), 2,4,6-tris(pyridyl-s-triazne (TPTZ), sulphanilamide, *N*-(1-naphthyl) ethylenediamine dihydrochloride, sodium nitroprusside, linoleic acid, R-phycoerythrin were acquired from Sigma-Aldrich (St. Louis, MO, USA). Neochlorogenic acid was purchased from Extrasynthese (Genay Cedex, France).

### 3.2. Extraction

The seeds (1 kg for each cultivar) were ground into flour with an IKA® All Basic mill (IKA Works Inc., Wilmington, NC, USA) and passed through a 60-mesh sieve. The flour was macerated with aqueous methanolic mixture (5 L, 80:20; v/v), at room temperature for fifteen days with occasional shaking. The process was repeated three times with same quantity of solvent mixture. The extracts obtained were combined, filtered through filter paper under vacuum and concentrated under reduced pressure on a rotary evaporator (model Q-344B-Quimis, Sao Paulo, Brazil) using a warm water bath (model Q-214M2-Quimis) at 37 °C–40 °C, to obtain a thick gummy mass, which was further dried in a desiccator and stored in air-tight vial till further use.

### 3.3. Total Phenolics and Condensed Tannins Contents

The content of total phenolic compounds in extracts was estimated using the Folin and Ciocalteau’s phenol reagent [39]. According to Heimler *et al.* [40] and Xu and Chang [41], gallic acid was used as a standard in this work and results were reported as mg gallic acid equivalents (GAE)/g. Condensed tannins (proanthocyanidins) were analyzed using acidified vanillin reagent [42] and results were expressed as mg catechin equivalents (CE)/g. 

### 3.4. RP-HPLC

For the RP-HPLC finger print analysis of phenolic compounds present in the extracts a Shimadzu system (Shimadzu Corp., Kyoto, Japan) consisting of two LC-10AD pumps, SCTL 10 A system controller, SPD-M 10 A photo-diode array detector, and a prepacked LUNA C 18 (4 × 259 mm, 5 μm, Phenomenex) was used. A flow rate 1 mL/min, and gradient elution of acetonitrile-water-acetic acid (5:93:2, v/v/v) [solvent A] and of acetonitrile-water-acetic acid (40:58:2, v/v/v) [solvent B], 0–50 min solvent B from 0% to 100% was used [43]. Concentration of sample dissolved in methanol was 10 mg/mL; injection volume 20 μL; separation of compounds was monitored at 280 and 320 nm. 

### 3.5. DPPH Radical Scavenging Activity

DPPH^•^ scavenging activity of cowpea extracts was evaluated according to the method described earlier [44]. The absorbance of the sample was measured using an UV 160 spectrophotometer, (Shimadzu, Kyoto, Japan) at 517 nm against ethanol blank. A negative control was taken after adding DPPH^•^ solution to 0.2 mL of the respective extraction solvent. Antiradical activity was calculated from equation:

$$Antiradical\;activity\;\%=(1-\frac{{Absorbance}_{sample}}{{Absorbance}_{control}})\times 100$$

Results were expressed as micromoles of Trolox equivalent per gram of extraxt (μmol Trolox/g) from the tests of the triplicate extracts using the calibration curve of Trolox. Linearity range of the calibration curve was 20 to 1,000 μM (r 0.99).

### 3.6. Ferric-Reducing Antioxidant Power (FRAP) Assay

The FRAP assay was performed as described by Benzie and Strain [45]. The sample solution analyzed was first properly diluted with deionized water to fit within the linearity range of Fe^2+^. FRAP value was expressed as mmol of Fe^2+^ equivalents per g of extract using the calibration curve of Fe^2+^. Linearity range of the calibration curve was 0.1 to 1.0 mM (*r* = 0.99).

### 3.7. Oxygen Radical-Absorbing Capacity (ORAC) Assay

Hydrophilic ORAC assay was carried out on a Gemini EM microplate spectrofluorometer (Molecular Devices, Sunnyvale, CA, USA), which was equipped with an incubator and wavelength adjustable fluorescence filters. The procedures were based on the previous reports [46,47]. The kinetics of the fluorescence was recorded immediately by the software SoftMax Pro (Molecular Devices). The final ORAC values were calculated using a linear equation between the trolox standards or sample concentration and net area under the fluorescein decay curve. The data were analyzed using Microsoft Excel (Microsoft, Roselle, IL, USA). The area under the curve (AUC) was calculated as: AUC = 0.5 + (R2/R1 + R3/R1 + R3/R1 + … + 0.5 Rn/R1), where R1 was the fluorescence reading at the initiation of the reaction and Rn was the last measurement. The net AUC was obtained by subtracting the AUC of the blank from that of a sample or standard. The ORAC value was calculated and expressed as mmol of TE/g of extract using the calibration curve of Trolox. The linearity range of the calibration curve was 5.0 to 50 mM (r 0.99). For each specific sample, triplicate extractions were analyzed.

### 3.8. Total Radical-Trapping Antioxidant Potential (TRAP) Assay

The TRAP was determined according to previously reported method based on the protection provided by antioxidants on the fluorescence decay of (*R*)-phycoerythrin (lag-phase) during a controlled peroxidation reaction [48]. The reaction mixture consisted of sample (120 µL), phosphate buffer (pH 7.4; 2.4 mL), bidistilled water (375 µL), diluted R-PE (30 µL) and 75 µL of 2,2-azobis(2-amidinopropane) dihydrochloride (ABAP); the reaction kinetics at 38 °C were recorded for 45 min (λ_ex_ 495 nm, λ_em_ 570 nm) by a LS-55 luminescence spectrometer (Perkin Elmer, Wellesley, MA, USA). TRAP values were calculated from the length of the lag-phase produced by extract and compared with that of Trolox and expressed as µmol of Trolox per g of extract.

### 3.9. Hydroxyl Radical Scavenging Activity

The hydroxyl radical scavenging capacity was measured using modified method as described previously [49]. The assay was performed by adding EDTA (0.1 mL; 1 mM), FeCl_3_ (0.01 mL; 10 mM), H_2_O_2_ (0.1 mL; 10 mM), deoxyribose (0.36 mL; 10 mM), 1.0 mL extract (20–200 µg/mL), phosphate buffer (0.33 mL; 50 mM; pH 7.4) and ascorbic acid (0.1 mL) in sequence. After ambient incubation, for 1 h, about 1.0 mL portion of the incubated mixture was mixed with 1.0 mL of TCA (10%, v/v) and 1.0 mL of (0.5%) TBA (in 0.025 M NaOH containing 0.025 M NaOH BHA) to develop the pink chromogen measured at 532 nm. The hydroxyl radical scavenging activity of the extract was reported as the percentage of inhibition of deoxyribose degradation and was calculated according to the following equation:

$$Inhibition\;(\%)\;=\;\frac{A_{0}\;-\;A_{1}}{A_{0}}\;\times \;100$$

where A_0_ - absorbance of the control and A_1_ - absorbance of the extract. Ascorbic acid was used as a positive control and results expressed as IC_50_.

### 3.10. Superoxide Radical Scavenging Activity

This activity was measured as described by Sabu and Ramadasan [50]. Test solutions of extract (20–200 µg/mL) were taken in a test tube. To this, reaction mixture consisting of sodium carbonate (1 mL; 50 mM), NBT (0.4 mL; 24 mM) and EDTA solutions (0.2 mL; 0.1 mM) were added to the test tube and immediate reading was taken at 560 nm. About 0.4 mL of hydroxylamine hydrochloride (1 mM) was added to initiate the reaction then reaction mixture was incubated at 25 °C for 15 min and reduction of NBT was measured at 560 nm. Ascorbic acid was used as the reference compound. Decreased absorbance of the reaction mixture indicates increased superoxide anion scavenging activity. The percentage of inhibition was calculated according to the following equation:

$$Inhibition\;(\%)\;=\;\frac{A_{0}\;-\;A_{1}}{A_{0}}\;\times \;100$$

where A_0_ - absorbance of the control and A_1_ - absorbance of the extract. Ascorbic acid was used as a positive control and results expressed as IC_50_.

### 3.11. Nitric Oxide Scavenging Activity

Nitric oxide scavenging activity was measured spectrophotometrically [51]. Sodium nitroprusside (5 mM) in phosphate buffered saline was mixed with different concentrations of the extract (3 mL; 20–200 µg/mL) dissolved in methanol and incubated at ambient conditions. After 30min, 1.5 mL of the incubated solution was removed and diluted with 1.5 mL of Griess reagent (1% sulphanilamide, 2% phosphoric acid and 0.1% *N*-(1-naphthyl)ethylenediamine dihydrochloride). The absorbance of the chromophore formed during diazotization of the nitrite with sulphanilamide and subsequent coupling with *N*-(1-naphthyl)ethylenediamine dihydrochloride was measured at 546 nm and percentage scavenging activity was measured with reference to standard by following equation:

$$Inhibition\;(\%)\;=\;\frac{A_{0}\;-\;A_{1}}{A_{0}}\;\times \;100$$

where A_0_ - absorbance of the control and A_1_ - absorbance of the extract. Ascorbic acid was used as a positive control and results expressed as IC_50_.

### 3.12. Antioxidant Activity in Linoleic Acid System

The antioxidant activity of sample extracts was determined following the method of Osawa and Namiki [52]. Sample extracts were added to a solution mixture of linoleic acid (0.13 mL), 99.8% ethanol (10 mL) and 0.2 M sodium phosphate buffer (pH 7.0, 10 mL). The total volume was adjusted to 25 mL with distilled water. The solution was incubated at 40 °C for 360 h and the degree of oxidation was measured according to the thiocyanate method [53] with 10 mL of ethanol (75%; v/v), 0.2 mL of an aqueous solution of ammonium thiocyanate (30%; m/v), 0.2 mL sample solution and 0.2 mL of ferrous chloride (FeCl_2_) solution (20 mM in 3.5% [m/v] HCl) being added sequentially. After 3 min of stirring, the absorption values of mixtures measured at 500 nm were taken as peroxide contents. A control was performed with linoleic acid but without the extracts. Synthetic antioxidants BHA and BHT were used as the positive controls. The percent inhibition of linoleic acid peroxidation to express antioxidative activity was calculated from the equation

$$Antioxidant\;activity\;(\%)\;=\;100\;-\;(\frac{\Delta {Absorbance}_{sample}\;after\;360\;h}{\Delta {Absorbance}_{control}\;after\;360\;h}\;\times \;100\;)$$

### 3.13. Statistical Analysis

All analyses were performed in triplicate and values expressed as the mean ± standard deviation. Data analysis was carried out using the analysis of variance and Tukey’s post-hoc test. 

## 4. Conclusions 

Although cowpea seeds are increasingly consumed as human food in Pakistan, the beneficial effects of their bioactive compounds remain largely unexplored. The assessment of antioxidant potential might be a fruitful approach for advocating them as nutraceuticals, in addition to them being potential protein and carbohydrate sources. The consumption of a processed cowpea would not only improve nutrient utilization, but also provide potential nutraceuticals for human health. It could therefore be concluded that cowpea could contribute significantly in the management and/or prevention of degenerative diseases associated with free radical damage, in addition to their traditional role of preventing protein malnutrition. 

## Figures and Tables

**Table 1:** Contents of total phenolics and condensed tannins (mg/g) of the extracts of cowpea seeds.

Contents	CP1	CP2	White star	SA dandy
Total phenolics	11.9 ± 0.2 ^c^	14.0 ± 0.1 ^b^	19.32 ± 0.1 ^a^	16.2 ± 0.1 ^b^
Condensed tannins	14.9 ± 0.2 ^c^	19.2 ± 0.4 ^b^	25.4 ± 0.4 ^a^	20.9 ± 0.6 ^b^

Mean values in the same row having different letters differ significantly (*p* < 0.05). CP1, CP2, White star and AS dandy are the names of cowpea cultivars.

**Table 2:** Content of individual phenolic compounds in the extracts (mg/g).

Compound	CP1	CP2	White star	SA dandy
**1**	0.033 ± 0.002 ^b^	0.029 ± 0.002 ^b^	0.060 ± 0.003 ^a^	0.057 ± 0.002 ^a^
**2** (neochlorogenic acid)	0.127 ± 0.006 ^c^	0.130 ± 0.006 ^c^	0.221 ± 0.010 ^a^	0.178 ± 0.009 ^b^
**3**	0.073 ± 0.003 ^c^	0.070 ± 0.003 ^c^	0.120 ± 0.005 ^a^	0.113 ± 0.005 ^b^
**4**	0.161 ± 0.008 ^c^	0.127 ± 0.006 ^d^	0.245 ± 0.012 ^a^	0.223 ± 0.010 ^b^
**5** (chlorogenic acid)	1.45 ± 0.07 ^d^	1.79 ± 0.09 ^c^	2.69 ± 0.13 ^a^	2.31 ± 0.12 ^b^
**6**	0.104 ± 0.005 ^c^	0.092 ± 0.003 ^d^	0.121 ± 0.007 ^a^	0.116 ± 0.005 ^b^
**7** (caffeic acid)	0.466 ± 0.022 ^d^	0.884 ± 0.042 ^a^	0.602 ± 0.031 ^b^	0.502 ± 0.028 ^c^
**8**	0.115 ± 0.007 ^c^	0.114 ± 0.005 ^c^	0.302 ± 0.013 ^a^	0.281 ± 0.013 ^b^
**9**	0.278 ± 0.012 ^d^	0.369 ± 0.019 ^c^	0.428 ± 0.020 ^a^	0.397 ± 0.020 ^b^
**10**	0.355 ± 0.015 ^c^	0.292 ± 0.014 ^d^	0.769 ± 0.035 ^a^	0.674 ± 0.031 ^b^

Mean values in the same row having different letters differ significantly (*p* < 0.05). Compounds **1**, **3**, **4**, and **6** were expressed as *p*-coumaric acid; compounds **8**, **9** and **10** as caffeic acid.

**Table 3:** Antioxidant activity of the methanolic extracts of cowpea seeds.

Assay	CP1	CP2	White star	SA dandy
DPPH (µmol Trolox/g)	25.1 ± 0.6 ^b^	27.9 ± 0.7 ^b^	32.5 ± 0.2 ^a^	28.2 ± 0.4 ^b^
FRAP (mmol Fe^2+^/g)	15.5 ± 0.2 ^b^	13.2 ± 0.4 ^b^	19.4 ± 0.2 ^a^	18.0 ± 0.6 ^a^
ORAC (µmol Trolox/g)	86.7 ± 1.2 ^b^	83.8 ± 1.0 ^b^	96.2 ± 0.9 ^a^	89.7 ± 1.4 ^b^
Inhibition of linoleic acid peroxidation (%)	88.1 ± 2.0 ^c^	93.4 ± 1.8 ^ab^	96.6 ± 2.3 ^a^	90.2 ± 1.5 ^bc^
TRAP (μmol Trolox/g)	65.6 ± 1.1 ^c^	73.0 ± 0.9 ^b^	87.3 ± 1.2 ^a^	77.6 ± 0.3 ^b^

Mean values in the same row having different letters differ significantly (*p* < 0.05).

**Table 4:** Antiradical activity [IC_50_ (μg/mL)] of the extracts of cowpea seeds.

Scavenging activity	CP1	CP2	White star	SA dandy
Against hydroxyl radical	92.4 ± 1.1 ^a^	84.3 ± 0.2 ^b^	80.6 ± 0.4 ^c^	86.5 ± 1.0 ^b^
Against nitric oxide radical	138 ± 2 ^a^	125 ± 1 ^b^	108 ± 0.4 ^d^	113 ± 1.0 ^c^
Against superoxide radical	112 ± 1 ^a^	103± 1 ^b^	91.2 ± 0.9 ^d^	97.0 ± 1.4 ^c^

Mean values in the same row having different letters differ significantly (*p* < 0.05).
